# Maintenance costs of serotiny in a variably serotinous pine: The role of water supply

**DOI:** 10.1371/journal.pone.0181648

**Published:** 2017-07-21

**Authors:** Ruth C. Martín-Sanz, Marta Callejas-Díaz, Jeanne Tonnabel, José M. Climent

**Affiliations:** 1 Department of Forest Ecology and Genetics, Forest Research Centre, National Institute for Agricultural and Food Research and Technology, Madrid, Spain; 2 Sustainable Forest Management Research Institute (University of Valladolid-National Institute for Agricultural and Food Research and Technology), Palencia, Spain; 3 Department of Ecology and Evolution, Biophore Building, University of Lausanne, Lausanne, Switzerland; Aristotle University of Thessaloniki, GREECE

## Abstract

Serotiny is an important adaptation for plants in fire-prone environments. However, different mechanisms also induce the opening of serotinous cones in the absence of fire in variably serotinous species. Xeriscence -cone opening driven by dry and hot conditions- is considered to be mediated only by the external environment, but endogenous factors could also play a significant role. Using the variably serotinous *Pinus halepensis* as our model species, we determined the effects of cone age and scales density in cone opening, and using in-situ and ex-situ manipulative experiments we investigated the role of water availability in the opening of serotinous cones. We hypothesized that loss of connection between the cones and the branch through the peduncles or the absence of water supply could induce a faster cone opening. Results showed that older cones lost more water and opened at lower temperatures, with no influence of scales density. Both field and chamber manipulative experiments (using paired cones of the same whorl) confirmed that water intake through the peduncles affected significantly the pace of cone opening, such that lack of water supply speeded up cone dehiscence. However, this was true for weakly serotinous provenances—more common in this species—, while highly serotinous provenances were indifferent to this effect in the field test. All our results support that cone serotiny in *P*. *halepensis* involves the allocation of water to the cones, which is highly consistent with the previously observed environmental effects. Importantly, the existence of maintenance costs of serotinous cones has strong implications on the effects of climate change in the resilience of natural populations, via modifications of the canopy seed banks and recruitment after stand-replacing fires. Moreover, evolutionary models for serotiny in *P*. *halepensis* must take into account the significant contribution of maintenance costs to the complex interaction between genotype and the environment.

## Introduction

The numerous drivers of global change are posing new challenges for plant species in many forest ecosystems worldwide (see, for example [1]). In the Mediterranean region, climate warming is already increasing the frequency and intensity of extreme climatic events such as heat waves and droughts [2–4]. Fire is an endemic characteristic of these areas, and thus plant species show several fire adaptive traits [5,6]. Nevertheless, forecast new climatic conditions may facilitate the spread of wildfires and increase their frequency and intensity [7,8], hampering post-fire regeneration and posing a potential strain on forests resilience.

Serotiny -i.e., the maintenance of closed cones in the canopy after seed ripening- is widely recognised as an adaptation to fire in many woody plants [3,9,10], although some authors claim that this trait should be considered an exaptation rather than an adaptation to fire [11]. However, different mechanisms of cone opening synchronize seed release with different environmental conditions [12–14]. The opening of pyriscent cones is prompted by higher temperatures caused by fires that melt their resins, while necriscent cones open with the death of their protective tissues, killed either by fire or due to plant senescence [12]. Moreover, xeriscent cones can open in dry and hot periods, thus releasing their seeds even in the absence of fire [15–17].

The duration of seed retention is highly variable both among species [5,18,19] and within and among populations of some species [20–22]. But, beyond the evidences of genetically-based variation in serotiny that might reflect local adaptation in some species [23], species with cones that eventually open without fire pose intriguing questions about the role of interacting genetic and environmental effects. While the fleshy living cones of some Angiosperms (*Banksia*, *Hakea* and *Leucadendron* as the best studied genera) are widely recognized to depend on the water supply of the bearing plant to remain closed [19,24,25], the lignified cones of conifers are generally regarded as merely dependent on the external conditions [14]. However, tree water status seems to affect the opening of cones both in *Cupressus* [26], and in *Pinus halepensis* Mill. [16] suggesting a possible competition for water between older and younger cones.

Phenotypic plasticity, or the ability of a genotype to generate different phenotypic values for a given trait under different environmental conditions, is an important strategy by which plants can maximize fitness in fluctuating environments [27,28]. The first experimental quantitative evidence of plasticity for serotiny in *P*. *halepensis* has shown that the number of highly serotinous trees is lower in drier environments [29] in accordance to previous field observations [16,17]. Furthermore, a strong negative allometric effect on serotiny has been found for *P*. *halepensis*, i.e. smaller or younger trees are fully serotinous, and degree of serotiny decreases with size [15,30,31]. This pattern has been also found in other species [32–34] and it is considered adaptive since this allows the maintenance of a big enough aerial seed bank at early reproductive stages. Importantly, the lower degree of serotiny at dry conditions would be expected if the only effects involved in the opening of serotinous cones of this species were exogenous, i.e. direct environmental effects, but the fastest loss of serotiny relative to tree size under cold conditions reveals allometric plasticity due to a combination of direct and indirect environmental effects [29]. In the same study, total cone number also affected negatively the proportion of serotinous cones beyond tree size and site conditions, suggesting that the resources available to the tree likely impose trade-offs of resource allocation limiting the amount of cones that a tree can maintain closed.

*Pinus halepensis* has a variable and intermediate level of serotiny that has been extensively studied in recent years [15,23,31 and many others]. However, the causes that trigger cone opening in the absence of fire are still unclear and are a key point of debate. Moreover, some authors ponder that *P*. *halepensis* has a dual strategy based on two different types of cones in the same individual: serotinous and not serotinous cones with different physical structure and anatomy [35,36]. Several studies consider only mechanical changes in serotinous cones mediated by external weather conditions as the main trigger [14], but other studies consider possible endogenous causes, such as the age of the cones [31] or the internal condition of the cones resulting from the balance of plant resources (water and perhaps other substances transferred through the peduncle [16]). The hypothesis of the latter study is based on field observations and on parallelism with other species, both gymnosperms [26] and angiosperms (see for example, [24,25]). Our own field observations indicated that serotinous cones of broken or dry branches open distinctly before cones of healthy similar branches of the same tree, suggesting an endogenous control of cone opening. Moreover, physical cone damage (e.g. due to squirrels or crossbills) can also provoke precocious cone opening while insects or herbivory can impede cone dehiscence.

Despite its likely importance, it is not known whether a physical connection between the cone and the supporting branch is even possible, thus allowing physiological exchanges between the tree and the cones. This was a key starting point for our work. Interestingly, the particularly thick peduncle (differentiating *P*. *halepensis* from its close relative *P*. *brutia* Ten. [37]), could be related to a more long-lasting conducting capacity in the first species. These two close related species show xeriscent cones, but despite the lack of direct quantitative comparisons, *P*. *brutia* is regarded to have lower reproductive precocity and lower investment in reproduction along with lower serotiny than *P*. *halepensis* [30,37]. Moreover, thick cone peduncles are not displayed by other serotinous pine species related to *P*. *halepensis* within the *Pinaster* section [38], such as *P*. *pinaster* Ait. and *P*. *canariensis* C. Sm.

Here, we investigate the role of water availability in the opening of serotinous cones on the Mediterranean *P*. *halepensis*, which could be related to maintenance costs of serotiny. For this purpose, we performed experiments combining in-situ and ex-situ manipulation of both plant to cone physical connection and water availability to the cones in *P*. *halepensis*. Our work hypothesis was that the vascular connection of the cones to the branch through the peduncles and the water intake affect serotinous cone opening in this species, i.e. loss of connection or an absence of water supply may induce a faster cone opening. Additionally, we assumed that removing vascular connection and water intake, thus modifying the internal water status of the cones, would be more critical at intermediate external conditions, while at extremely hot and dry outer environment all cones will open irrespectively of their water supply.

Our primary objective was to investigate whether the internal environment affects the opening of serotinous cones once all other effects were removed, including allometric effects (total number of serotinous cones and tree size) and genetic effects among individuals and provenances. We previously determined whether older cones opened at lower temperature as shown by Tapias et al. [31] and whether we could distinguish two different types of cones (serotinous and not serotinous) that could be related to physical differences as the scales density [36].

## Material & methods

Almost all female cones from *P*. *halepensis* individuals remain attached to the branches after ripening—either open or closed—. This characteristic is relevant to study our hypothesis and makes *P*. *halepensis* a good model species for our purpose.

Since smaller/younger trees are more serotinous [29,31], young individuals combine a high availability of serotinous cones and an easier access from the ground with just the help of standard telescopic pruning scissors.

### Laboratory screening experiment

#### Plant material and protocol

The aim of this experiment was to determine the effect of cone age on cone opening [31] and to investigate the existence of two different types of cones -serotinous and non-serotinous–which could be related to physical differences [35,36].

We collected serotinous cones of *P*. *halepensis* from young trees (aged < 20 yr) in two different planted stands in Central Spain (Guadalajara -GUA- and Madrid -VAL-, Table 1). These two sites are public lands not protected in any way. For the type of sampling performed no special permit was required, although we had verbal permission from forest managers anyway. The field study did not involve protected or endangered species. All cones were initially stored in a cold chamber with controlled temperature. We randomly selected 15 trees in each stand to assess tree age, by ring-counting in cores extracted at the tree base (height 0.1 m) with an increment borer. The tree stands are even aged, estimation of age in 15 trees provides a reliable estimation of tree age at the stand level.

**Table 1:** Description of the experimental sites and sampling at each site.

Plant stand	Latitude	Longitude	Altitude (m)	Age of trees (years)	P (mm)	Ps (mm)	T (°C)	MMTW (°C)	MMTC (°C)	Sampling type	N. of samplings
**GUA**	40°41’16”N	3°6’38”W	840	15	596	69	12.3	22.5	3.8	Cone pairs	30
**VAL**	40°9’18”N	3°41’8”W	672	17	491	62	13.7	24.5	5.0	Individual cones	161
**Common garden**											
**ALT**	39°49’29”N	0°34’22”W	605	17	417	81	14.4	22.9	7.9	Cone pairs	69
**OLM**	40°38’42”N	3°26’44”W	731	17	475	62	13.6	24.3	4.9	Cone pairs	87

P, annual rainfall; Ps, rainfall of the three warmest months (June, July and August); T, mean annual temperature; MMTW, mean maximum temperature of the three warmest months; MMTC, mean minimum temperature of the coldest month (all previous variables were representative of the period 1951–1999, from [39]).

First, we determined the age of each cone by dendro-chronology of the insertion branch disc following the protocol by Tapias et al. [31]. Then, we introduced individual cones in a chamber where both temperature and humidity were controlled. Temperature was increased from 36 to 60°C while relative humidity was gradually decreased from 25 to 4% (see Table A in S1 Appendix). Due to chamber space limitation, we performed this process seven times with 24 cones in each test cycle. Cone opening was recorded using a webcam in time-lapse format. The videos were visualized to obtain the opening time of each cone, thus enabling to retrieve values of temperature and humidity at the time of cone opening (i.e. combination of temperature and humidity that triggered cone opening). Determining cone weight at the time of their opening would have provided information on the possible loss of water prior to cone opening. However, to maintain the controlled conditions in the chamber, we discarded obtaining these data. After the experiment, we also calculated the accumulated temperature as the heat sum suffered by each cone inside the chamber until opening.

We further obtained the dry weight of five scales from the central part of each cone after oven-drying during 24 hours at 60°C. We additionally calculated scales volume by measuring the hydrostatic thrust of the submerged scales in a water vase and finally estimated the scales density dividing scales volume by dry weight. Moisture content of each cone after the experiment was calculated by comparing its weight before and after the experiment by using Eq 1:

$$H=\frac{BW-AW}{AW}*100$$ (1)

Where BW is the weight of the cones before the experiment and AW is their weight after the experiment.

We measured cone weight after the experiment immediately after removing the cones from the chamber to prevent moisture changes.

#### Data analysis

Based on individual cones, we used Pearson’s correlations to investigate the relationship among opening temperature of the cones and the other experimental variables (i.e. age of the cones, scales density and cone moisture after the experiment). Subsequently, we analysed the effect of these variables on the opening accumulated temperature of the cones using linear mixed models (LMM) with test cycle as a random factor (Eq 2):

Accumulatedtemperature∼Age+Scalesdensity+Moisturecontent+(1|Testcycle) (2)

**Fig 1:**
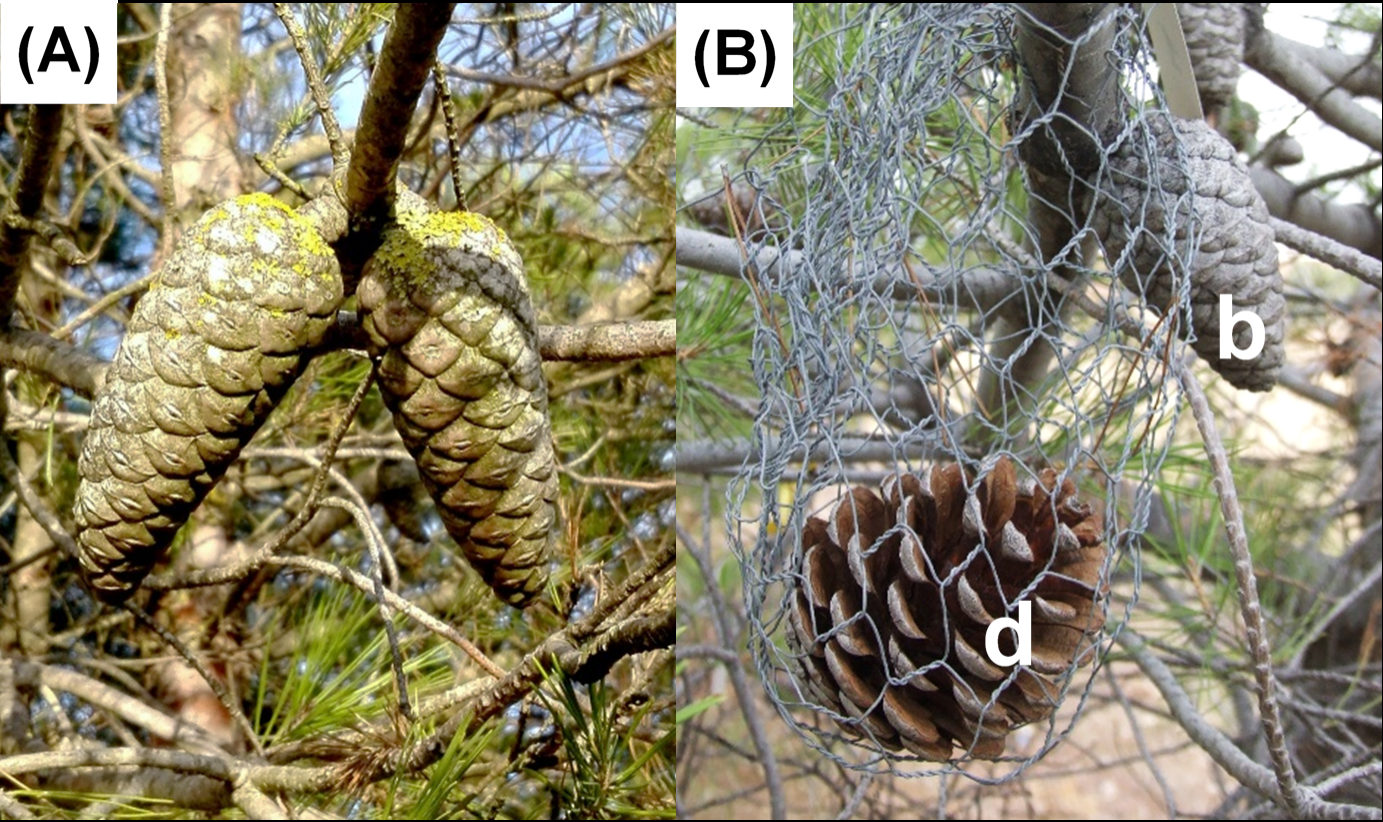
Description of the in situ manipulative experiment. (A) Pair of closed serotinous cones of the same whorl of *Pinus halepensis*. The thick and long peduncles characteristic of this species are easily distinguishable. (B) One of the possible cases that can be observed in the experiment: ‘Positive’, i.e., detached cone opened earlier than its branched pair; b, branched cone -control-; d, detached cone.

Due to the results from this preliminary experiment, indicating the absence of two different types of cones in Aleppo pine (serotinous and non-serotinous) and the significant effect of cone age, we decided to follow our investigation by comparing paired cones from the same whorl, i.e. two cones of the same age from the same branch (Fig 1A).

### Manipulating water availability ex situ

#### Plant material and protocol

**Fig 2:**
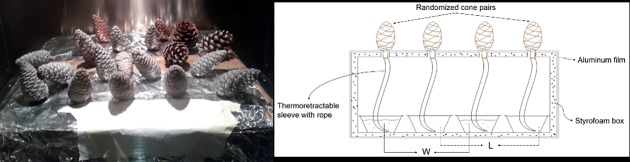
Manipulating water availability in the *ex situ* experiment. (A) Photograph of the styrofoam box inside the chamber. An aluminum film cover was used to further increase isolation and prevent water heating. (B) Scheme of the experiment inside the styrofoam box. W, watered cones; L, waterless cones.

In this experiment, we sought to investigate the effect of water supply through cone peduncle on cone opening. We collected pairs of serotinous cones from the same whorl in the same two planted stands described in the first experiment (Table 1). All cones were initially stored in a cold chamber with controlled temperature before the experiment. This second laboratory experiment was carried out in the controlled chamber previously described, accounting for temperature and relative humidity while providing or not water supply to the cones. The rationale behind is that the putative effect of water supply might have an effect solely at intermediate temperatures, while at moderately high temperatures (ca. 60°C) all cones would open irrespectively of their water supply. Both cones of the pair were subjected to a controlled cycle of increasing temperature (from 24 to 60°C) and decreasing relative humidity (from 40 to 4%) inside the chamber (Table B in S1 Appendix). Cone opening was video-monitored with the same method used in the first experiment. We supplied external water to one cone of the pair (watered cones) while keeping the other cone dry (waterless cones). Water supply was provided through a thermoretractable sleeve with a rope inside, everything sealed within a styrofoam box except the cones (Fig 2). This way we prevented both water evaporation and heating during the experiment. Cones were randomly distributed in the box. We also calculated the moisture content following Eq 1 and the accumulated temperature of each cone in this experiment as we did in the laboratory screening experiment.

#### Data analysis

We used binomial generalized linear mixed models (GLMM) for analysing the treatment effect between watered and waterless cones of each pair on their probability to open. We included treatment and accumulated temperature for cone opening as fixed factors, and cone within pair as random factor (Eq 3):

Opening∼Treatment+Accumulatedtemperature+(1|Pair:Cone),family=binomial (3)

In this experiment all the cones eventually opened, thus the binary cone opening variable was coded as follows: cones which opened before their pair and cones which opened later. Finally, to check whether the waterless cones lost or not more water than the watered cones at the end of the ex situ experiment, moisture content of the cones after the experiment was also evaluated using linear mixed models (LMM) with treatment as fixed factor, and pair and test cycle as random factors (Eq 4):

Moisturecontent∼Treatment+(1|Pair)+(1|Testcycle) (4)

### Manipulating tree to cone physical connection *in situ*

#### Plant material and protocol

This field manipulative experiment was carried out in February and April 2014, when trees were 17 years old, in a *P*. *halepensis* common garden trial established in 1997 (see [40] for details). All sites of the common garden are composed of trees coming from the same natural populations of origin (hereafter, provenances). We chose two sites (out of the six sites established in eastern and central Spain) because of their similar favourable environmental conditions for this species ([41], ALT and OLM in Table 1). Other sites characterized by harsher climatic conditions–cold and dry- and therefore potentially limiting growth and survival of this species were excluded due to a lower availability of serotinous cones. As already said, cone serotiny is plastic in such a way that serotiny is much lower at harsher sites [29]. We considered the two sites as replicates of the experiment rather than contrasting environments to reveal putative plasticity in the process of cone opening. They also were accessible and controlled, facilitating a long-lasting manipulative experiment. To account for variation among individuals, we selected highly reproductive and highly serotinous trees, marking three branches per tree with a pair of serotinous cones at each branch (Fig 1A). In total, we selected 84 pairs of serotinous cones from 29 trees at OLM site and 51 pairs of serotinous cones from 21 trees at ALT site. For each pair, one of the cones was excised by the peduncle as close to the branch as possible and placed in a wire basket hanged at the same place on the branch (detached cones, hereafter). Its ‘twin’ cone was kept untouched still physically connected to the branch (branched cones hereafter–control-; Fig 1). Therefore, both cones experienced exactly the same external conditions, allowing us to discard this effect in our results. To avoid water loss, both cuts at the branch and the peduncle of the removed cone were immediately stoppered with pruning mastic (Lac Balsam, Compo S.A.).

We monitored the experiment by recording each cone status (open or close) every few months during the first year, starting the tenth week after its establishment. The following years, we limited ourselves to one observation before summer and one after it, since we expected summer to be the period driving the greater variations in cone opening. We performed more frequent observations and for a longer period at OLM site, where the experiment was ended in September 2016 (lasting 125 weeks), whereas at ALT site we terminated the experiment in April 2015 (lasting 60 weeks).

The need for a minimum number of serotinous cones per tree obliged a non-random selection of the provenances, although individual tree selection was otherwise random. Due to this different representation of the provenances between sites (only eight provenances were common to both of them), accounting for this factor strongly restricted our analysis. Therefore, in order to include the genetic effect in our study, we did a global analysis with both site together and another with just OLM site, where we had more observations and a better representation of provenances. We created a new categorical variable that we called ‘group of provenances’ for using data from ALT and OLM sites together. This new variable grouped provenances with similar behavior in reproduction, serotiny and growth in two highly contrasted groups (Northeast and Southwest). Southwest group comprises highly serotinous provenances with a marked reproductive precocity and allocation, while provenances of the Northeast group have lower serotiny and reproductive allocation [40, 42]. This division is coherent with the weak geographical structure so far shown based in neutral markers (Ruiz-Daniels et al. in prep.). Thus, we firstly used the complete dataset for the two sites with the factor ‘group of provenances’ and secondly, we restricted ourselves to the trees that belonged to the common provenances at both sites using in this case the factor ‘provenances’. Our results were robust with these two options. However, due to a lower amount of data when using the eight common provenances, the models showed convergence problems so we finally decided to use the complete dataset. In addition, we also studied the genetic effects among provenances using just the site with more prolonged observations–OLM site-. In this case, we also measured basal diameter and the number of serotinous cones counted in 15 seconds [42] for each of the trees to account for allometric effects and putative competition for resources [16].

#### Data analysis

In order to investigate the treatment effect (cone detached or not) on the probability of cone opening at both sites–ALT and OLM-, we used binomial generalized linear mixed models (GLMM) while accounting for data structure as paired serotinous cones. We used treatment, group of provenances, site and their interactions as fixed factors and individual tree as a random factor (Eq 5).

Opening∼Treatment*Groupofprovenances*Site+(1|Tree),family=binomial (5)

To explore the treatment effect on the probability of cone opening at OLM site, we used binomial generalized linear models (GLM) with treatment, provenance and their interaction as fixed factors. We also included basal diameter and the number of serotinous cones as covariates (Eq 6).

Opening∼Treatment*Provenance+Basaldiameter+Serotiny,family=binomial (6)

However, neither of these two variables displayed significant effects on cone opening; therefore both covariates were eliminated from the final models.

Finally, we performed a non-parametric McNemar’s test considering cone pairs as twin samples to evaluate the evolution of the treatment effect on cone opening at OLM site. This non-parametric statistical test is used to compare paired proportions or discordance of two dichotomous responses when pairs are independent [43]. In our case, we compared the frequencies of four possible cases (Fig 1): “Close”, both cones of the pair remained closed; “Positive”, the detached cone opened before its branched pair; “Negative”, the branched cone opened earlier; “Open”, both cones opened between observations.

All the mixed models in this work were implemented in R 3.3.2. [44] using the lme4 package [45]. We evaluated fixed terms significance of linear and generalized linear mixed models (Eqs 2, 3, 4 and 5) using means of likelihood ratio test (LRT) between the full model and reduced models without each variable, showing in the results section the chi-square, degrees of freedom and *P*-value for those likelihood ratio tests. To select the most parsimonious model for each experiment, we compared the Akaike’s Information Criterion (AIC) of models with the most appropriate variables as random factors in each equation, as well as with a non-randomized model. We also ranked GLM models using AIC, selecting the model with the lowest value.

## Results

**Fig 3:**
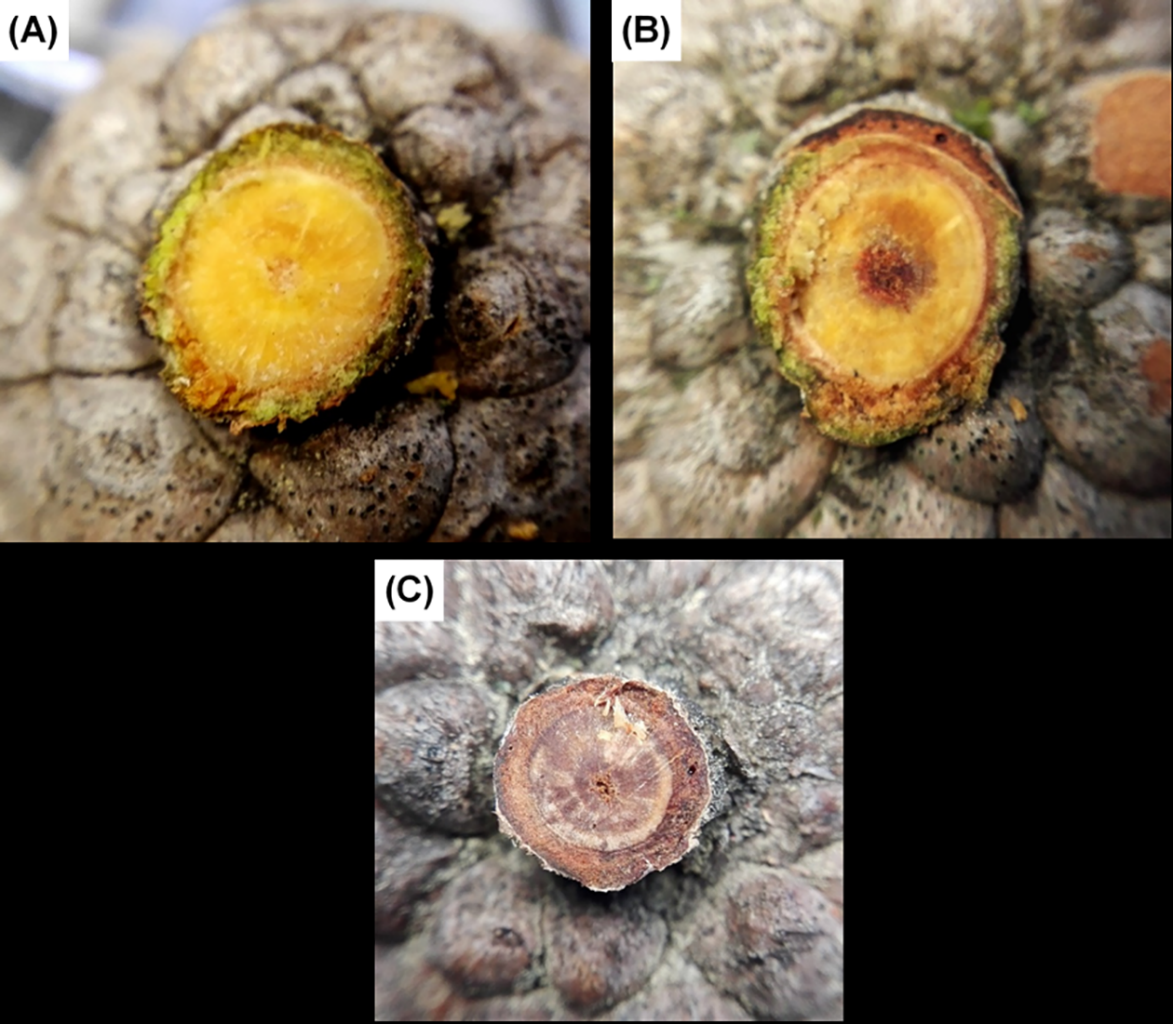
**Peduncle sections pertaining to serotinous cones of > 5 years from *Pinus halepensis* (A, B) and *P*. *brutia* (C)**. Xylem of (A) peduncle is entirely sapwood; (B) xylem has a typical incipient heartwood starting from the pith and (C) is entirely heartwood. (A) and (B) peduncles show living cortical tissues (phloem and cortical parenchyma), but not (C). 99% of *P*. *halepensis* cones sampled in this study were in the ‘(A)’ status.

All *P*. *halepensis* cones used in this study that were detached from the tree (ca. 540) had peduncles showing sapwood xylem, clear-coloured and wet when cut, and living cortical tissues (Fig 3A). Only two of the examined cones showed peduncles with partial heartwood formation—easily distinguishable by its reddish brown colour (Fig 3B and 3C)—and were eliminated from the following experiments.

### Laboratory screening experiment

**Fig 4:**
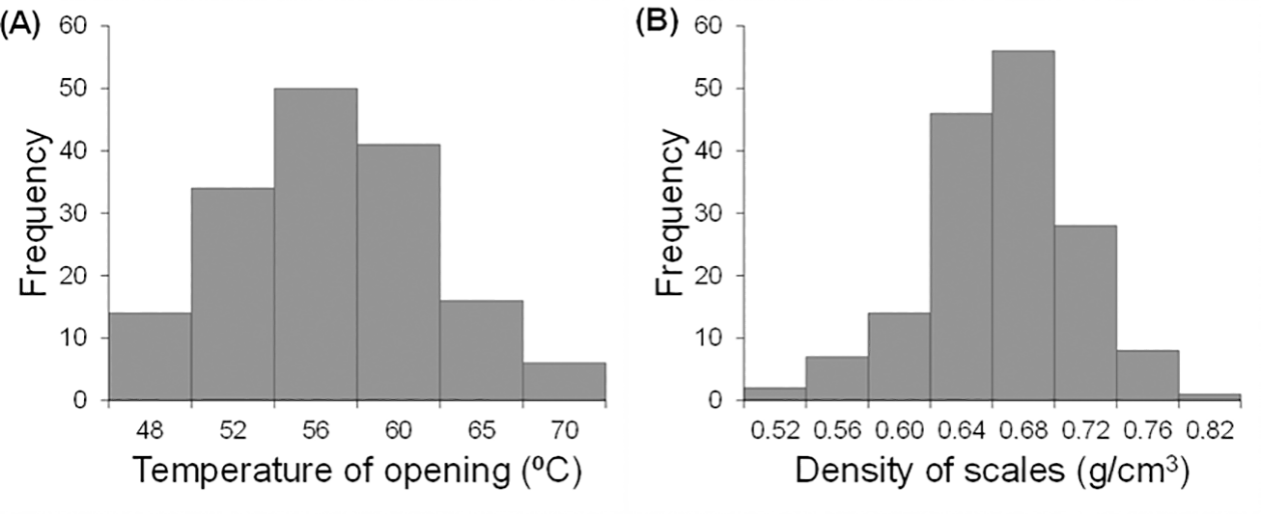
Frequency distribution for two variables in the laboratory screening experiment. (A) Frequency distribution for temperature of cone opening and (B) for density of the scales of the cones.

Confirming our hypotheses, older cones opened at lower accumulated temperature (r = -0.33; *P* < 0.0001) and displayed lower moisture content after the experiment than younger cones (r = -0.24; *P* = 0.005). However, older and younger cones did not differ in scale density. Scale density was uncorrelated with their respective opening temperature, but it was related to cone moisture, with the densest cones showing a lower moisture content after the experiment (r = -0.19; *P* = 0.023). Moreover, we found a rather continuous variation for both cone opening temperature and scales density (Fig 4A and 4B).

We found a significant negative effect of cone age on cone opening accumulated temperature (χ^2^ = 7.85, DF = 1, *P* = 0.005), but scale density and moisture content did not affect significantly the cone opening temperature (χ^2^ = 1.00, DF = 1, *P* = 0.317 and χ^2^ = 1.07, DF = 1, *P* = 0.301, respectively; Table C in S1 Appendix).

### Manipulating water availability ex situ

The cones that received water supply through their peduncles opened after their waterless twin cone (χ^2^ = 17.74, DF = 1, *P* < 0.0001). Meaningfully, the waterless cones lost significantly more water throughout the experiment than their watered pairs (χ^2^ = 33.13, DF = 1, *P* < 0.0001). The accumulated temperature had a negative effect in cone opening (χ^2^ = 4.71, DF = 1, *P* = 0.023; Table C in S1 Appendix) showing a decreasing effect of the treatment along the experiment. Unlike in the field experiment, all cones opened in response to the controlled extreme conditions. Although 14.8% of cone pairs opened almost at the same time, and 18.5% of the “wet” cones opened before its “dry” pair, the majority of watered cones (66.7%) opened -as expected- after their dry pair.

### Manipulating tree to cone physical connection in situ

**Fig 5:**
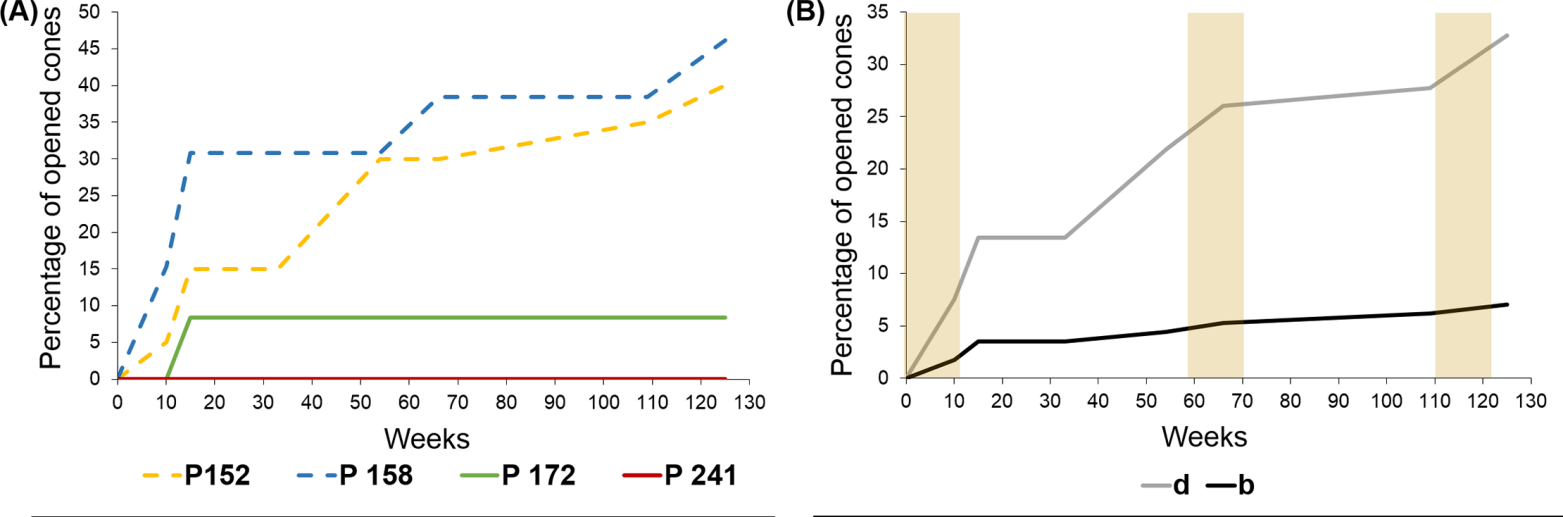
Cone opening at OLM site by provenance and treatment. (A) Percentage of cone opening for some provenances with contrasting behaviour pertaining to the two different provenance groups. Provenances P 152 and P 158 are part of the Northeast group; provenances P 172 and P 241 are part of the Southwest group. This plot illustrates the significant treatment by provenance interaction found at this site. (B) Percentage of cone opening during the experiment at OLM site, by treatment. Brown shadows indicate summer seasons. b, branched cones -control-; d, detached cones.

The results for both sites (Eq 5) showed that the detached cones exhibited a strongly significant higher probability of opening than their respective paired cones that remained attached to the tree (χ^2^ = 49.09, DF = 1, *P* < 0.0001; Table C in S1 Appendix). Site was slightly significant on cone opening (χ^2^ = 4.34, DF = 1, *P* = 0.037), as well as group of provenances (χ^2^ = 6.37, DF = 1, *P* = 0.012), with trees from the Northeast group showing faster opening compared to the Southwest group (Fig 5A). The estimated opening percentage for the Northeast group varied between 38 to 60% at ALT and OLM sites respectively, while for the Southwest group, the percentage of cone opening was lower at both sites (17 to 34%, respectively). However, none of the interactions were significant, not even the interaction between treatment and provenance group or between provenance group and site (χ^2^ = 0.22, DF = 1, *P* = 0.637, χ^2^ = 3.24, DF = 1, *P* = 0.072, respectively).

The GLM restricted to OLM site (Eq 6) showed also a highly significant effect of the treatment (F_1,170_ = 32.11, *P* < 0.0001), as well as a significant effect of the provenance (F_12,158_ = 30.62, *P* = 0.002). The interaction between treatment and provenance was also significant (F_12,146_ = 23.12, *P* = 0.023; Table D in S1 Appendix).

**Fig 6:**
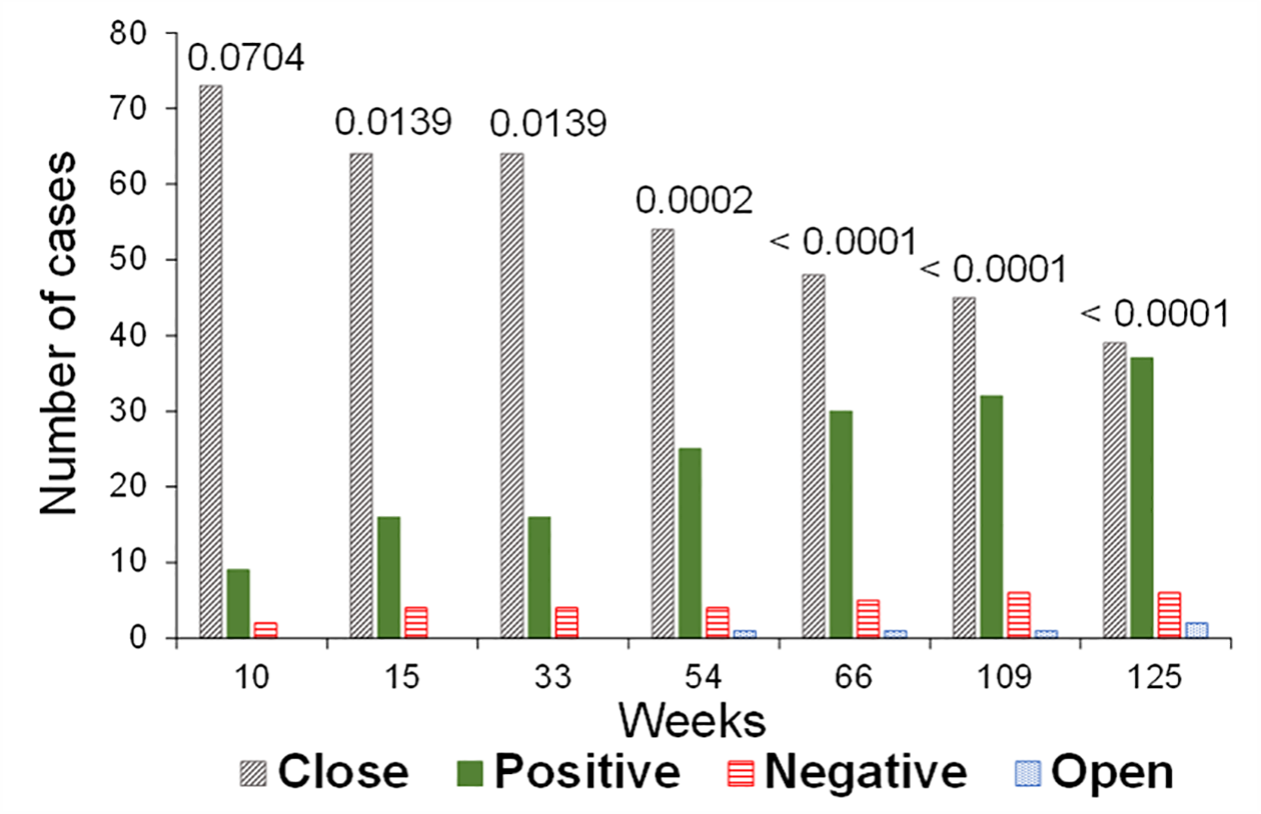
Progress of the field manipulative experiment at OLM site from 10 to 125 weeks after its setting up. Categories meaning is the following: ‘Close’, both cones of the pair remained closed; ‘Positive’, the detached cone opened earlier; ‘Negative’, the attached cone opened earlier; ‘Open’, both cones opened between observations. Numbers in the bars correspond to the significance of McNemar’s test.

At OLM site—where longer observations were performed—, the focal cones opened progressively since the observation onset, but still 72% of them remained closed after 31 months. Additionally, only 36% of cones that remained closed corresponded to detached cones. As expected, the greater changes in the percentage of open cones occurred during the summers (Fig 5B). The non-parametric test (McNemar’s) for OLM site showed a significant treatment effect (i.e. attached vs. detached cones) since the onset of the experiment (χ^2^ = 3.27, DF = 1, two-tailed *P* = 0.070), continuously increasing the strength of the treatment effect until the last observation in September 2016 (χ^2^ = 20.93, DF = 1, two-tailed *P* < 0.0001; Fig 6).

## Discussion

Hereby, we provide the first direct experimental evidence that endogenous conditions play a critical role in the maintenance of closed serotinous cones in a variably serotinous species.

We found a continuous variation both in the opening temperature and in the scales density of the cones. This result do not support the existence of two different types of cones as was previously exposed [35,36], at least in our sampled material. Moreover, our results confirmed that older cones opened at lower temperatures [31] and they also lost more water during the experiment. These points to a physical degradation of older cones, probably due to the weathering of cuticles and other protective compounds, like reported in other pine species [46,47]. Also, the apparent decrease in cones’ resilience for older cones combined with the observation that cones of different ages shared similar scales density make us reject the hypothesis that the higher serotiny of younger trees [29,31] could be due to physical differences in the first cone cohorts.

Contrary to what was previously admitted as a general rule for pines, our study reveals that the peduncle xylem of cones in *P*. *halepensis* remains sapwood for a long time, therefore enabling the interaction between the tree and the cones (Fig 3). Thick peduncles occur in some other serotinous pines, like *Pinus coulteri* D. Don [48], while in many others—including all other serotinous pines of the subsection *Pinaster*, the closest relatives to *P*. *halepensis*—the peduncle does not enlarge and transforms into heartwood. By contrast, other conifers, like Cupressus or Sequoia are well known to display living cones with sapwood peduncles [26]. Unfortunately, we lack a broad enough comparative study on this feature across the genus *Pinus*, although this is a cornerstone for investigating maintenance costs of serotinous cones.

Our laboratory experiment aimed at confirming the role of water supply in a reverse way than the subsequently field manipulative experiment; this is, supplying water through the peduncle to half of the cones while keeping the rest dry. As hypothesised, cones lacking water supply opened significantly earlier than their watered twins did. In addition, waterless cones lost more water than their watered pairs during the experiment. However, most cones opened in a quite narrow temperature interval (from 47°C to 52°C), i.e. the treatment effect between the two-paired cones was shown as different timing at the same temperature, not as different opening temperature. Furthermore, as expected, the highly significant effect of the treatment decreased with accumulated temperature. Meaningfully, the effect of watering the cone through the peduncle was detectable only at the lower range of temperature x time, while higher temperatures (higher than those occurring naturally in the field in the absence of fire) provoked an abrupt opening that blurred the effect of water intake. It is important to remember that, for obvious practical reasons, the programmed heating schedule in the chamber did not mimic the conditions of the field experiment, where the process was much slower. Importantly, the relative water content of the cones after the experiment was significantly affected by the treatment, with lower water content in the waterless cones, further confirming that our water deprivation treatment was actually affecting the internal water condition of the cones. These results are highly consistent with the assumptions and previous hypotheses indicating the relevant role of water status of the cone in its probability to open or remained closed, once that other sources of variation were eliminated.

The field manipulative experiment showed that the cones which were detached from the branch opened significantly earlier than the branched cones, at both experimental sites. Our simple but straightforward experiment seems to be the first of its kind. Unlike previous experiments, the experimental design including the use of cone pairs virtually eliminates all microenvironmental variation between the branched and the detached cones. Moreover, the variation due to cone age and branch variability were also eliminated by this design. This field experiment was particularly informative at OLM site due to its longer term. Interestingly, in this site the treatment significance showed by the non-parametric test increased over time (Fig 6). Cone opening shot up during or after each summer season throughout the experiment, especially for the detached cones. This demonstrates that the xeriscent condition of *P*. *halepensis* cones (i.e. only considering the external environment, [14]) is clearly modified by the internal environment. In our field conditions, this endogenous effect via peduncle caused a 5-fold reduction in the probability of opening after three summers (from 33% down to 7%). These results clearly fulfilled our hypotheses highlighting the relevance of the physiological condition of the trees in cone serotiny in *P*. *halepensis* [16,29], similarly to other species with well supported maintenance costs of serotiny like Cupressus and several Proteaceae [19,24–26].

Meaningfully, the field experiment also confirmed the strong genetic effect on cone serotiny in *P*. *halepensis*, already evidenced in previous works [21–23]. As expected, provenances of the Northeast group (less serotinous and reproductive) were more likely to open their cones than provenances of the Southwest group, either analysing the genetic effect directly with the provenances or separating the provenances into two groups by their similar behaviour. The interaction between treatment and the genetic effect was only significant at OLM site, where we used the factor ‘provenance’, likely because a higher number of provenances was represented at this site due to our experimental design. Results of the interaction between treatment and genetic effect conform with previous description of differences between provenances: the most serotinous provenances maintained their cones closed for much longer regardless of treatment—i.e. even the detached cones remained closed—while cones from less serotinous provenances opened in a greater proportion, with a high treatment effect (detached cones opened earlier). We think that the stronger sealing of cones of highly serotinous populations (as revealed, for example by cone-opening tests in warm water, [31]) is probably an overpowering effect compared to the water status of the cone. These marked genetic differences associated to the provenance are also probably hiding the expected allometric effects of tree size and the total number of serotinous cones in the crown interacting with our manipulative treatment. These ontogenetic and allometric effects are backed by previous works but its quantification is indeed complex [49]. Applying more intense manipulative experiments to genetically homogeneous individuals grown under stressful conditions could eventually help disentangling allometric effects on serotiny from other concomitant effects, including maintenance costs. One can also note that further work should confirm that water allocation to the serotinous cones entail a cost at the scale of the tree. Experiments investigating re-allocation of resources could help highlight such a cost: for example, by assuming that tree physiology is partitioned between different branches, one could examined allocation to vegetative tissues and serotiny duration in branches where most cones would have been harvested compared to branches that remain intact.

All these experimental evidences suggest that the observed plasticity in the degree of serotiny in *P*. *halepensis* (lower serotiny in cold-dry, growth-limiting conditions, [29]) is due to both endogenous and exogenous effects. Thus, naming *P*. *halepensis* a strictly xeriscent species as recognized currently (cone opening directly triggered by dry and/or hot air conditions, [14]) appears to be inaccurate.

## Conclusions

Our results highlight that the duration of serotiny in *P*. *halepensis* involves the allocation of water to the cones and thus suggest that maintenance cost of serotiny can be more important than previously appreciated.

Considering the effect of age on cone opening (older cones are more prompt to open) and that our trees were fairly young, the higher serotiny of younger trees is not due to a higher inherent serotiny of the first cone cohorts but, very likely, due to a physical degradation (weathering) as the cone remains in the crown. Once that age and other sources of variation are kept invariable thanks to the use of paired cones from the same whorl, both field manipulative experiments and chamber experiments confirmed that water intake through the peduncles affected significantly the pace of cone opening, such that lack of water supply speeded up cone dehiscence. This was true for most provenances ranging from weakly to moderately serotinous, while trees from the few highly serotinous provenances virtually did not respond at all to our treatments, likely because of a stronger sealing of the cone scales.

We think our results open the field for more precise ecophysiological studies, since we did not measure actual water transfer via xylem or other possible interchange via phloem in the connected cones. The existence of maintenance costs of serotinous cones has strong implications on the effects of climate change (combining more frequent severe droughts and wildfires) in the resilience of natural populations, via modifications of the canopy seed banks and recruitment after stand-replacing fires. Moreover, evolutionary models for serotiny in *P*. *halepensis* must take into account the significant contribution of maintenance costs to the complex interaction between genotype and the environment.

## Supporting information

S1 Appendix This appendix contains all Supporting Tables (A, B, C and D). **Table A.** Cycle of controlled temperature and relative humidity used in the screening laboratory experiment with individual cones. **Table B.** Cycle of controlled temperature and relative humidity used in the manipulating water availability ex situ experiment with pairs of cones from the same whorl. **Table C.** Results of linear and generalized linear mixed models for the different experiments. **Table D.** Summary of the selection process of generalized linear models and results of the chosen GLM for the field manipulative experiment carried out at OLM site.
(PDF)

S2 Appendix This appendix contains all the databases used for this work. **Table A.** Database with all the variables used in the screening laboratory experiment. **Table B.** Database with the variables used in the ‘manipulating tree to cone physical connection in situ’ experiment for both sites. **Table C.** Database with the variables used in the ‘manipulating tree to cone physical connection in situ’ experiment only for OLM site. **Table D.** Database with all the variables used in the ‘manipulating water availability ex situ’ experiment. (EXCEL file).
(XLSX)
