# Supplementary material for: Maintenance costs of serotiny in a variably serotinous pine: The role of water supply
Source: PLoS One. 2017 Jul 21;12(7):e0181648. doi: 10.1371/journal.pone.0181648 (PMC5521817; doi:10.1371/journal.pone.0181648)
Supplement: S1 Appendix — Table A. Cycle of controlled temperature and relative humidity used in the screening laboratory experiment with individual cones. Table B. Cycle of controlled temperature and relative humidity used in the manipulating water availability ex situ experiment with pairs of cones from the same whorl. Table C. Results of linear and generalized linear mixed models for the different experiments. Table D. Summary of the selection process of generalized linear models and results of the chosen GLM for the field manipulative experiment carried out at OLM site. (PDF) [file pone.0181648.s001.pdf]

S1 Appendix

Table A. Cycle of controlled temperature and relative humidity used in the screening laboratory experiment with individual cones.

Time (min)	Temperature (℃)	Relative humidity (%)
5	36	25
60	36	25
5	36 – 40	25 – 20
120	40	20
5	40 – 44	20 – 15
120	44	15
5	48 – 52	10 – 8
300	48	10
5	48 – 52	10 – 8
240	52	8
5	52 – 56	6 – 4
300	56	6
5	56 – 60	6 – 4
240	60	4

Table B. Cycle of controlled temperature and relative humidity used in the manipulating water availability ex situ experiment with pairs of cones from the same whorl.

Time (min)	Temperature (℃)	Relative humidity (%)
15	0 – 24	40
120	24	40
5	24 – 28	40 – 35
120	28	35
5	28 – 32	35 – 30
240	32	30
5	32 – 36	30 – 25
240	36	25
5	36 – 40	25 – 20
360	40	20
5	40 – 44	20 – 15
360	44	15
5	44 – 48	15 – 10
360	48	10
5	48 – 52	10 – 8
360	52	8
5	52 – 56	8 – 6
240	56	6
5	56 – 60	6 – 4
240	60	4

Table C. Results of linear and generalized linear mixed models for the different experiments. Models were fitted excluding the relevant terms. Akaike's Information Criterion (AIC), and model degrees of freedom (DF) are given for each model. Chi-square statistic (Chisq), degrees of freedom (df) and *P*-value are given for likelihood ratio tests between the model with and without the correspondence variable. Variance and standard deviation for the random effects of the chosen models are given.

**Model**	**AIC**	**DF**	**Chisq**	**df**	***P*-value**	**Var.**	**Std. Dev.**
**Eq. 2**							
Age + Dens + H	3021.2	6					
Age	*3019.2*	*4*	7.85	1	**0.005****		
Dens	3028.2	4	1.00	1	0.317 n.s.		
H	3025.9	4	1.07	1	0.301 n.s.		
Random effects: Test cycle						1.042e+07	3.228e+03
Residual						1.684e+08	1.298e+04
**Eq. 3**							
Treat + Accum.T	*60.5*	*4*					
Treat * Accum.T	62.5	5	0.03	1	0.860 n.s.		
Treat	63.2	3	17.74	1	**<0.0001*****		
Accum.T	76.2	3	4.71	1	**0.023***		
Random effects: Pair:Cone						0.291	0.540
**Eq. 4**							
Null	243.9	4					
Treat	*212.8*	*5*	33.13	1	**<0.0001*****		
Random effects: Pair						1.287	1.135
Test cycle						1.355	1.164
Residual						1.382	1.176
**Eq. 5**							
Treat + Group + Site	*245.6*	*5*					
Treat * Group * Site	248.3	9	1.98	1	0.159		
Treat * Group	249.9	5	0.22	1	0.637		
Treat * Site	252.0	5	0.00	1	0.994		
Group * Site	292.8	5	3.24	1	0.072		
Treat	252.2	3	49.09	1	**<0.0001*****		
Group	295.9	3	6.37	1	**0.012***		
Site	297.7	3	4.34	1	**0.037***		
Random effects: Tree						0.479	0.692

Accum.T = Accumulated temperature; Treat = Treatment; Group = Group of provenances.
AIC and DF of the chosen model for each experiment are in italics.
Significant *P*-values for the fixed effects are in bold case.

Table D. Summary of the selection process of generalized linear models and results of the chosen GLM for the field manipulative experiment carried out at OLM site. Akaike's Information Criterion (AIC) and degrees of freedom (DF) are given for each model. Degrees of freedom of each parameter (df), deviance (Dev.), residual degrees of freedom (Res.df), residual deviance (Res. Dev.) and *P*-value of the ANOVA table are given for each explanatory variable of the chosen model.

**Model**	**AIC**	**DF**	df	Dev.	Res. df	Res. Dev.	*P*-value
**Eq. 6**							
Treat * Prov + Db + Ser	168.4	28					
Treat * Prov + Db	167.9	27					
Treat * Prov + Ser	167.3	27					
Treat * Prov	167.9	26	12	23.12	146	115.89	**0.023***
Treat + Prov	*167.0*	*14*					
Treat	173.6	2	1	32.11	170	169.64	**<0.0001*****
Prov	203.2	13	12	30.62	158	139.01	**0.002****
Null	203.7	1			171	201.75	

Treat = Treatment; Prov = Provenances.
The chosen model is in italics.
Significant *P*-values are in bold case.
